# Efficient Hydrogen Production from Ammonia Using Ru Nanoparticles on Ce-Based Metal–Organic Framework (MOF)-Derived CeO_2_ with Oxygen Vacancies

**DOI:** 10.3390/molecules30112301

**Published:** 2025-05-23

**Authors:** Wenying Wu, Wenhao Yao, Yitong Liu, Senliang Xi, Teng Zhang

**Affiliations:** Key Laboratory of Cluster Science Ministry of Education, Beijing Key Laboratory of Photoelectronic/Electrophotonic Conversion Materials, Advanced Research Institute of Multidisciplinary Science, School of Chemistry and Chemical Engineering, Beijing Institute of Technology, Beijing 100081, China; wwy15933833065@163.com (W.W.); ywh200012@163.com (W.Y.); m18735721812_1@163.com (Y.L.)

**Keywords:** Ru catalyst, ammonia decomposition, oxygen vacancy, metal–organic frameworks

## Abstract

Ammonia is a promising hydrogen storage material because it is easy to store and decompose into CO_X_-free hydrogen. A Ru-based catalyst exhibits good catalytic performance in ammonia decomposition, and enhancing the interaction between the Ru atoms and the support is an important way to further improve its catalytic activity. In this study, CeO_2_ was prepared by calcination using a cerium-based metal–organic framework (MOF) as the precursor, and the number of oxygen vacancies on the surface of CeO_2_ was regulated by hydrogen reduction. The XPS and Raman results showed that abundant oxygen vacancies were formed on the surface of these CeO_2_, and their number increased with an increase in the reduction time. The Ru/CeO_2_-4 h catalyst, using CeO_2_ reduced for 4 h as the support, exhibited good catalytic activity in ammonia decomposition, reaching 98.9% ammonia conversion and 39.74 mmol g_cat_^−1^ min^−1^ hydrogen yield under the condition of GHSV = 36,000 mL g_cat_^−1^ h^−1^ at 500 °C. The XAFS results demonstrated that Ru was stably anchored with oxygen vacancies on the surface of CeO_2_ via Ru-O-Ce bonds. Density functional theory calculations further showed that these bondings lower the reaction energy barrier for N-H bond cleavage, thereby significantly enhancing the catalytic activity.

## 1. Introduction

Hydrogen energy storage and transportation is an important link restricting the development of hydrogen energy. The flammable and explosive characteristics of hydrogen make its transportation a difficult problem [1,2]. Ammonia is considered to be a promising hydrogen storage medium, which has the advantages of easy liquefaction (25 °C, 8.6 bar), high-volume hydrogen density (120 kg cm^−3^), low manufacturing cost, and itself being a carbon free fuel [1,3]. The properties of ammonia are conducive to the overall improvement of production, storage, and transportation infrastructure [4,5,6].

The decomposition reaction of ammonia is an endothermic process, manifested as follows:

2NH_3_ (g) → N_2_ (g) + 3H_2_ (g)  ΔH = 91.2 kJ mol^−1^ (1)

To achieve the complete conversion of ammonia, the decomposition temperature usually needs to be maintained over 800 °C, which would consume a large amount of energy. Thus, developing novelly efficient catalysts to reduce the energy consumption is very important for the industrial application of ammonia decomposition to hydrogen [7]. In recent years, the development of ammonia decomposition catalysts has made great progress [8,9,10,11]. Fe, Co, Ni, Ru, and other metal catalysts have been widely researched, providing a variety of options for the development of ammonia decomposition technology. Among these metals, Ru is the most effective active center due to its excellent catalytic activity at low and medium temperatures [12,13,14,15]. Meanwhile, the selection of the carrier is also very important. As reported in previous work, the strong metal–support interaction (SMSI) between Ru and the carrier could further enhance the catalytic activity of the Ru catalyst for NH_3_ decomposition [16,17,18,19,20,21,22].

Metal oxides, such as CeO_2_ [23,24,25], MgO [26,27], La_2_O_3_ [28], Al_2_O_3_ [29,30,31], ZrO_2_ [32,33], are widely used as carriers for Ru catalysts in the ammonia decomposition process due to their surface acidity/basicity, surface oxygen vacancy, reoxidation properties, and the interaction between metal and support. Among these carriers, CeO_2_ showed higher NH_3_ decomposition catalytic activity than Ru/Al_2_O_3_ due to the strong SMSI and electronic modification of Ru active sites by CeO_2_ [34]. Hu et al. [35] loaded Ru single atoms onto cerium oxide nanospheres (CeO_2_-Nss) prepared by an improved colloidal deposition method and cerium oxide nanorods (CeO_2_-NRs) prepared by the hydrothermal method. N_2_ and H_2_ on CeO_2_-Ns and CeO_2_-NR catalysts are more easily desorbed than MgO-supported catalysts prepared by other methods. According to the research, Ru/CeO_2_ has great potential as the ammonia decomposition catalyst.

For the last few years, the application of the metal–organic framework (MOF) in catalyst preparation has been widely studied [36]. The MOF structure combines inorganic metals and organic linkers with a high surface area, diversity of assemblies, and uniform porosity. These advantages give the MOF the potential to be the catalyst, catalyst support, or precursor in many chemical reactions [37]. There have been numerous reports on the preparation of CeO_2_ using MOF structures. Sivan et al. [38] reported a Ce-BTC-derived Ru/CeO_2_ catalyst with highly dispersed Ru, and it shows excellent and more stable performance in NH_3_ synthesis. He et al. [39] reported that the high-porosity Ru/CeO_2_ catalyst prepared with Ce-UiO-66 improved the catalytic performance of CO_2_ methanation compared with the CeO_2_-supported Ru catalyst prepared by the traditional method. Chen et al. [40] used CeO_2_ obtained by pyrolysis of Ce-MOF for toluene combustion; when compared with CeO_2_ prepared by the precipitation method, MOF-CeO_2_ exhibits a better catalytic activity due to its structure and abundant oxygen vacancy. According to the research, CeO_2_ derived from Ce-MOF could exhibit superior surface properties to support the Ru metal, and this strategy is a potential path to prepare high-performance ammonia decomposition catalysts.

In this study, the Ce-BPDC was synthesized as the precursor, followed by calcination in air and reduction in hydrogen atmosphere to obtain CeO_2_ with abundant oxygen vacancies. The number of oxygen vacancies on the surface of CeO_2_ were adjusted by controlling the reduction time. Then, the Ru catalysts supported on these CeO_2_ were prepared by impregnation, and their catalytic performances on ammonia decomposition were evaluated. Furthermore, these catalysts were comprehensively characterized to explore the relationship between the structure and catalytic properties, as well as the reaction mechanism.

## 2. Results and Discussion

### 2.1. Structural Characterization of Carrier and Catalyst

The characterization results of Ce-BPDC are consistent with the results in the literature (see Supporting Information for detailed description). The PXRD results of CeO_2_-t obtained by calcining Ce-BPDC at 500 °C and then reducing in the H_2_/Ar atmosphere for different times (t = 0, 0.5, 1, 1.5, 2, 3, 4 h) are given in Figure S4. The pattern of the CeO_2_ standard sample showed diffraction peaks at 28.5, 33.1, 47.5, 56.3, 59.1, 69.4, 76.7, 79.1, and 87.4, corresponding with a face-centered cubic phase of the CeO_2_ fluorite structure. Compared with that of CeO_2_, the diffraction peaks of CeO_2_-t shifted toward a higher angle, which could be attributed to the transformation of Ce^4+^ to Ce^3+^ during the calcination process of Ce-BPDC [41]. The N_2_ adsorption/desorption isotherms of CeO_2_-t are given in Figure S5. The specific surface area of cerium oxide obtained by the calcination of Ce-BPDC decreased significantly from 1502 m^2^ g^−1^ to about 45 m^2^ g^−1^. After loading Ru, the specific surface area of the catalyst did not change significantly. The morphology of CeO_2_-t was characterized by SEM and TEM (Figures S6 and S7). The results show that the morphology of CeO_2_-t after calcination was basically similar to that of the Ce-BPDC precursor, while its crystal particle size decreases.

**Figure 1:**
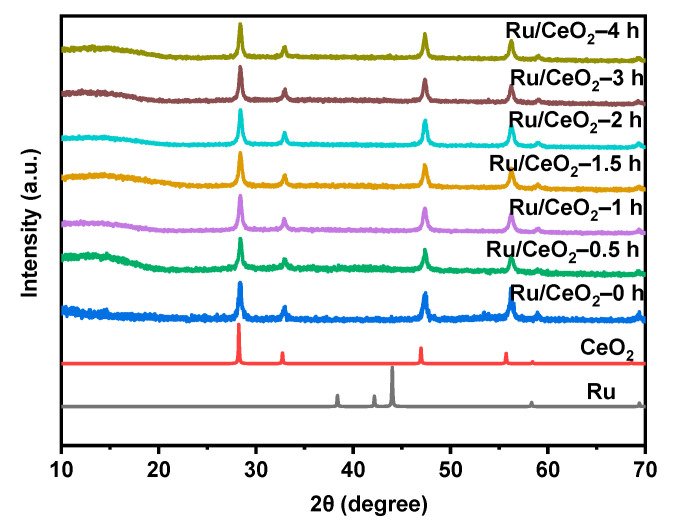
The PXRD profile of the Ru/CeO_2_-t.

The Ru/CeO_2_-t catalysts were prepared by the impregnation method with RuCl_3_, and their PXRD patterns are shown in Figure 1. It was found that the face-centered cubic phase of CeO_2_ fluorite structure was retained, and no characteristic diffraction peak of Ru was observed, indicating that Ru was highly dispersed on the surface of the support [42]. The Ru content in these catalysts was characterized through Inductively Coupled Plasma Optical Emission Spectrometry (ICP-OES). The results show that the Ru loading of Ru/CeO_2_-t (t = 0, 0.5, 1, 1.5, 2, 3, 4 h) was 4.29, 4.42, 4.34, 4.46, 4.20, 4.32, and 4.19 wt%, respectively. In order to have a deeper understanding of the catalyst microstructure and the dispersion of Ru on the CeO_2_ support, detailed observations were made using HRTEM. The lattice stripes of Ru and CeO_2_ can be observed in Figure S8. The spacing of 0.31 nm corresponds to the (111) face of CeO_2_. After doping Ru, a small number of Ru nanoparticles was observed on the surface of CeO_2_, and its lattice spacing was about 0.21 nm, corresponding to the (101) face of Ru substance. The EDS (Figure S8) results show that Ru was evenly dispersed on the surface of CeO_2_-t. The particle size of Ru in the catalyst Ru/CeO_2_-t (t = 0, 0.5, 1, 1.5, 2, 3, 4 h) was mainly concentrated between 1.5 and 4 nm (Figure S9).

### 2.2. Comparison of Catalytic Performance of Ammonia Decomposition to Hydrogen

The ammonia decomposition performance of the catalyst was tested in a self-built fixed-bed reactor (Figure S10), where 0.05 g catalyst was mixed with 2.95 g quartz sand and then transferred to a reactor equipped with quartz cotton. By injecting 50% NH_3_/Ar gas, the system temperature was adjusted to the corresponding reaction temperature. The catalyst was evaluated at a certain gas hourly space velocity (GHSV) in the temperature range of 375–500 °C. The detailed procedures are listed in the Supporting Information.

Under the GHSV condition of 12,000 mL g_cat_^−1^ h^−1^, the catalytic activities of Ru/CeO_2_-t and Ru/CeO_2_-C (Ru supported on the commercial CeO_2_) catalysts in NH_3_ decomposition were compared and analyzed, as presented in Table 1. It was found that, compared with Ru/CeO_2_-C, the catalytic performance of the MOF-derived CeO_2_-supported Ru catalyst significantly improved. In addition, the ammonia decomposition performance increased significantly with the increase in the reaction temperature. Meanwhile, the catalytic activity also increased on increasing the reduction time, during which the catalytic activity of Ru/CeO_2_-4 h is the highest. The ammonia conversion rate of Ru/CeO_2_-4 h reached 98.38% at 475 °C and 100% at 500 °C, respectively. In order to evaluate the practical application potential of Ru/CeO_2_-4 h catalyst, the ammonia decomposition performance at high GHSV (36,000 mL g_cat_^−1^ h^−1^) was evaluated. And the results are shown in Figure S11. It can be seen that Ru/CeO_2_-4 h still maintained a high catalytic activity, and the ammonia conversion rate was up to 97.04% at 475 °C and 98.9% at 500 °C, respectively. The hydrogen production rate was calculated according to the ammonia conversion of the catalyst (described in Section 3.3). Under the condition of GHSV = 36,000 mL g_cat_^−1^ h^−1^, the hydrogen production rate of Ru/CeO_2_-4 h was 38.99 mmol g_cat_^−1^ min^−1^ at 475 °C and 39.74 mmol g_cat_^−1^ min^−1^ at 500 °C, respectively. Under similar conditions, the synthesized Ru/CeO_2_-t catalyst was compared with the reported Ru catalysts, as shown in Table S2. It is worth noting that the catalytic activity of Ru/CeO_2_-4 h remained in the first echelon.

In order to evaluate the long-term stability of the catalyst, the Ru/CeO_2_-4 h catalyst was subjected to a 50 h stability test under GHSV = 36,000 mL g_cat_^−1^ h^−1^. The results are shown in Figure S12. No catalytic performance attenuation was observed after the 50 h test, which demonstrated that Ru/CeO_2_-4 h had excellent catalytic stability in the ammonia decomposition reaction.

### 2.3. Surface Chemical State of the Carrier and the Catalyst

It has been reported that hydrogen can react with lattice oxygen (O_L_) in CeO_2_ to form H_2_O, while inducing the reduction of Ce^4+^ to Ce^3+^ and generating oxygen vacancies (O_V_) [43]. The existence of O_V_ can enhance the SMSI between Ru and CeO_2_ support, and perhaps, this is the reason that Ru/CeO_2_-4 h exhibits excellent catalytic activity and stability. To systematically investigate the existence of O_V_ on the surface of CeO_2_-t, Raman spectroscopy and the XPS test were performed.

**Figure 2:**
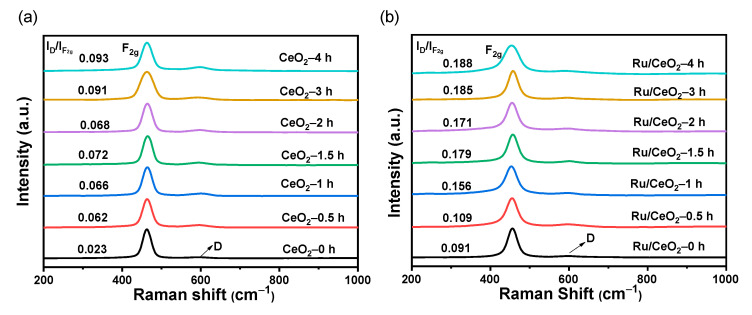
Raman spectra of various supports (**a**) and catalysts (**b**).

O_V_ can cause lattice distortions that create new characteristic peaks in the Raman spectrum or change the position and intensity of existing peaks [44]. Thus, Raman spectroscopy was used to reveal the defect location of the samples in this work, and the results are shown in Figure 2. The Raman peaks of CeO_2_-t and Ru/CeO_2_-t at 456 and 605 cm^−1^ can be attributed to the octahedral symmetric tensile vibration mode (F_2g_) and the defect induction mode (D) [45]. Since the D-mode peak is caused by the presence of Ce^3+^, the intensity ratio of the D-peak to the F_2g_ peak (I_D_/I_F2g_) can be used to reflect the relative concentration of O_V_ in CeO_2_ and Ru/CeO_2_ [46]. The results are given in Table S3. The I_D_/I_F2g_ value increased on increasing the reduction time, indicating that more O_V_ were formed on the surface of CeO_2_. The Raman spectrum of CeO_2_-C is shown in Figure S13. It was found that CeO_2_ prepared from the Ce-BPDC precursor had greater reducibility in a H_2_/Ar atmosphere than commercial CeO_2_, resulting in more O_V_ generation during the reduction process.

**Figure 3:**
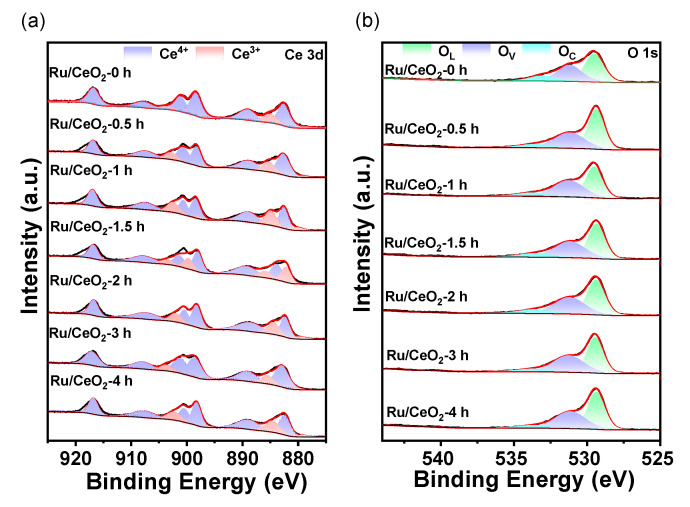
The (**a**) Ce 3d and (**b**) O 1s XPS profiles of Ru/CeO_2_-t.

In addition, the surface electronic states and chemical compositions of Ru/CeO_2_-t and the carrier CeO_2_ were studied by XPS. Ru 3p XPS measurements were performed to study the Ru valence states (Figure S14), which exhibited characteristic peaks of Ru^0^ (462.2 eV) and Ru^4+^ (465.2 eV). The different chemical valence states of Ru on these samples may be due to the charge transfer between CeO_2_ carriers and Ru nanoparticles. In order to verify this, the Ce 3d spectra were recorded. The Ce 3d spectra of CeO_2_-t are shown in Figure S15, and the Ce 3d spectra of Ru/CeO_2_-t are shown in Figure 3a, which were divided into 10 groups due to the hybridization of Ce 4f orbitals with O 2p valence bands [47]. The six peaks at 882.4, 889.4, 898.4, 901, 907.3, and 916.9 eV are attributed to the Ce^4+^ species, while the other four peaks at 881.4, 885.4, 899, and 903.6 eV are attributed to the Ce^3+^ species [48]. Each cerium cation is coordinated by eight oxygen anions, and due to the electronic structure of cerium, charge is reversibly transferred between Ce^4+^ and Ce^3+^ [49]. Thus, the emergence of Ce^3+^ species is usually accompanied by the formation of O_V_ on the CeO_2_ surface [50]. The concentration of O_V_ can be inferred from the relative atomic ratio of Ce^3+^/(Ce^4+^ + Ce^3+^), as shown in Table S4. It can be observed that the concentration of O_V_ on the surface of CeO_2_-t and Ru/CeO_2_-t increased with an increase in the reduction time, which is consistent with the Raman results. Among these catalysts, the Ce^3+^/(Ce^4+^ + Ce^3+^) ratio of Ru/CeO_2_-4 h was the highest, indicating that the most O_V_ was generated in Ru/CeO_2_-4 h, which accorded with the test results of the catalyst performance.

To further confirm the variation law of O_V_, the O 1s XPS spectra of Ru/CeO_2_-t catalysts were collected (Figure 3b). The peak at 529.1–529.6 eV corresponds to the O_L_, the peak at 531.1 eV corresponds to O_V_, and the peak observed at 533.4 eV is attributed to chemisorbed oxygen (O_C_) in CeO_2_ [51]. The number of O_V_ can be quantified according to the ratio of O_V_/(O_L_ + O_C_ + O_V_) [52]. The results are shown in Table S4. It decreased in the order of Ru/CeO_2_-4 h > Ru/CeO_2_-3 h > Ru/CeO_2_-2 h > Ru/CeO_2_-1.5 h > Ru/CeO_2_-1 h > Ru/CeO_2_-0.5 h, indicating that the number of O_V_ at Ru/CeO_2_-4 h was the highest. The concentration of O_V_ on the CeO_2_-t surface showed the same trend. The results were consistent with the Raman results and the Ce 3d XPS spectra.

Figure S16 presents the Ce 3d and O 1s XPS spectra of hydrogen-reduced commercial CeO_2_, with the quantified Ce^3+^/(Ce^3+^ + Ce^4+^) and O_V_/(O_V_ + O_L_ + O_C_) ratios summarized in Table S5. The results (Tables S4 and S5) demonstrate that CeO_2_ derived from Ce-BPDC displayed a superior reducibility. This result is consistent with the Raman result.

According to the literature, the surface basicity of the catalyst is conducive to ammonia decomposition; usually, the stronger the surface basicity, the higher the activity [53]. CO_2_-TPD was used to characterize the distribution of the surface basicity of the catalyst, and the results are shown in Figure S17. The number and intensity of basic sites can be determined according to the area and location of the desorption peaks. A certain number of weakly basic sites, moderately strong basic sites, and strong basic sites appeared in these catalysts. The area above 500 °C was a strong basicity site; the basicity strength increased with the increase in the reduction time. The desorption amount of CO_2_ on the surface of Ru/CeO_2_-4 h was the largest. From the above analysis, it can be concluded that the density of strong basic sites of Ru/CeO_2_-4 h was the largest, which is also in good agreement with the ammonia decomposition activity of the catalyst. As an electron donor, O_V_ increases the electron density of the adjacent metal site and enhances the Lewis basic site. On the surface of CeO_2_, oxygen ions near the O_V_ can act as Lewis base sites. The increase in O_V_ could increase the basicity sites on the surface of CeO_2_ to a certain extent [54]. Therefore, the concentration of O_V_ on the surface of Ru/CeO_2_-4 h catalyst is the highest. This was consistent with the Raman and XPS results.

**Figure 4:**
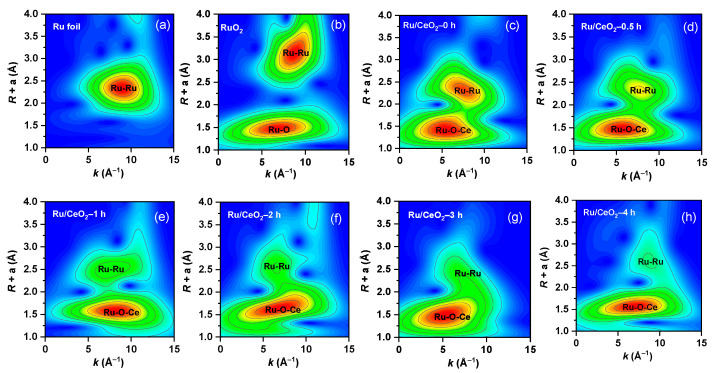
The WT-EXAFS of the Ru K-edge of Ru/CeO_2_-t, (**a**) Ru foil, (**b**) RuO_2_, (**c**–**h**) Ru/CeO_2_-t (t = 0, 0.5, 1, 2, 3, 4 h).

To further clarify the electronic states and coordination environment evolution of Ru species, we conducted Ru K-edge X-ray absorption fine structure (XAFS) tests on the Ru/CeO_2_-t (t = 0, 0.5, 1, 2, 3, 4 h) catalysts and used Ru foil (Ru^0^) and RuO_2_ (Ru^4+^) as reference standards. As shown in Figure S18a, the absorption edge positions of all the Ru/CeO_2_-t samples were similar to those of the RuO_2_ standard samples, confirming that Ru mainly existed in the +4 valence state (Ru^4+^). Figure S18b shows the Fourier transform Ru K-edge extended X-ray absorption fine structure (EXAFS) curves of all the samples. The figure shows two different peaks, 1.41 Å (the first shell) and 2.43 Å (the second shell), corresponding to Ru-O and Ru-Ru coordination, respectively. Then, a wavelet transform (WT) analysis was conducted on the EXAFS data, thereby further exploring in detail the contributions of different coordination shells to the Ru/CeO_2_-t EXAFS signal. In Figure 4, two main maximum intensities can be observed, which belong to the first coordination shell of Ru-O-Ce and the second coordination shell of Ru-Ru, respectively. The results show that Ru was stably anchored on the carrier surface through the Ru-O-Ce bond, and it was found that, with the increase in the reduction time, Ru-Ru gradually weakened, while the strength of the Ru-O-Ce bond increased. This further indicates that the interaction between Ru and the carrier increases with an increase in the reduction time.

### 2.4. Reaction Mechanism

To gain a deeper understanding of the O_V_ impact on ammonia decomposition, we conducted density functional theory (DFT) calculations and developed three reaction models: CeO_2_, Ru/CeO_2_, and Ru/CeO_2_-O_V_ (Figure S19). Figures S21 and S22 illustrate the reaction process of ammonia on the catalyst surface. The NH_3_ decomposition reaction follows a well-defined pathway: initially, NH_3_ is adsorbed onto the catalyst surface; subsequently, it undergoes a gradual dehydrogenation process; finally, N_2_ and H_2_ are generated and released from the surface.

**Figure 5:**
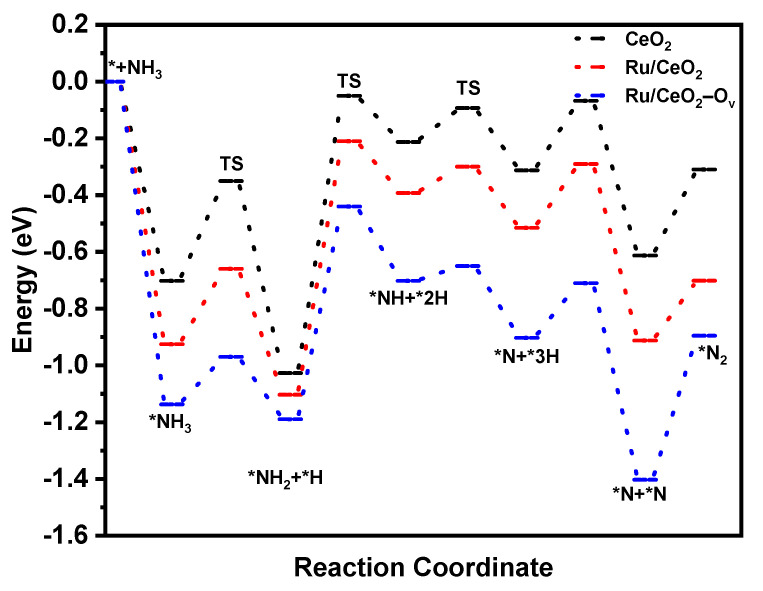
Potential energy diagram of NH_3_ dehydrogenation on the surface of CeO_2_, Ru/CeO_2_, and Ru/CeO_2_-O_V_ models.

As shown in Figure 5, the dehydrogenation potential energy of Ru/CeO_2_-O_V_ was lower than that of CeO_2_ and Ru/CeO_2_, which is due to the stronger adsorption of NH_3_ by this catalyst. It can be seen from the figure that the breaking of the N-H bond is the rate-determining step of the reaction. The reaction energy barrier required for the N-H cleavage of the Ru/CeO_2_-O_V_ system is lower than that of Ru/CeO_2_ and CeO_2_, thereby significantly improving the catalytic activity. Furthermore, Figure S20 compares the adsorption energy data of NH_3_ on the three models. The results show that the Ru/CeO_2_-O_V_ model had the highest adsorption intensity for NH_3_, reaching 1.021 eV, while the adsorption energies of the Ru/CeO_2_ and CeO_2_ models were 0.813 eV and 0.605 eV, respectively. This discovery clearly indicates that the presence of oxygen vacancies can reduce the reaction energy barrier for N-H bond breaking and significantly enhance the adsorption capacity of the catalyst for NH_3_. This phenomenon is due to the strong interaction between the metal and the carrier, which helps to improve the catalytic performance.

## 3. Experimental Section

### 3.1. Materials

Ammonium cerium nitrate (Ce(NH_4_)_2_(NO_3_)_6_, ≥98%), 4-4’ diphenyl dicarboxylic acid (H_2_BPDC, 99%), N, N-dimethylformamide (C_3_H_7_NO, DMF, ≥99.5%), acetone (C_3_H_6_O, ≥99.5%), anhydrous ethanol (C_2_H_5_OH, ≥99.7%), ruthenium trichloride (RuCl_3_, ≥99.9%) purchased from Aladdin Co., Ltd., and deionized water for laboratory use.

### 3.2. Catalyst Preparation

Ce-BPDC was synthesized by an improved hydrothermal method. The detailed preparation process is in the Supporting Information. After the successful synthesis of Ce-BPDC, it was calcined at 500 °C for 5 h to obtain CeO_2_. The CeO_2_ was reduced in the H_2_/Ar atmosphere for different durations (t = 0, 0.5, 1, 1.5, 2, 3, 4 h; 0 means the CeO_2_ sample was not reduced) at 500 °C, and the obtained samples were recorded as CeO_2_-t. The detailed preparation process of the carrier can be found in the Supporting Information. Briefly, 1 g of CeO_2_-t (t = 0, 0.5, 1, 1.5, 2, 3, 4 h) and 0.108 g (0.52 mmol) of RuCl_3_, respectively, were weighed and mixed in different glass bottles. Subsequently, 15 mL of deionized water was added to each vial and stirred on a mixing table for 8 h to obtain a gray solution. The solution was washed three times with deionized water, and the resulting precipitate was dried overnight in an 80 °C oven and finally allowed to yield the Ru catalyst. The experimental amount of Ru for each catalyst was 5 wt%. The resulting sample was named as the Ru/CeO_2_-t (t = 0, 0.5, 1, 1.5, 2, 3, 4 h) catalyst.

### 3.3. Catalyst Activity Evaluation

The hydrogen production evaluation process of ammonia decomposition catalyst was carried out in a fixed-bed reactor. Typically, 0.05 g of catalyst (40–60 mesh) was fully mixed with 2.95 g of quartz sand (40–60 mesh) and then transferred to a reactor equipped with quartz cotton. Before the activity test, the catalyst was heated to 200 °C with a ramp rate of 5 °C/min in 5% H_2_/Ar (50 mL/min) and reduced at 200 °C for 2 h. After switching to 50% NH_3_/Ar, the system temperature was adjusted to the corresponding reaction temperature. The catalyst was evaluated at a certain gas hourly space velocity (GHSV) in the temperature range of 325–600 °C at atmospheric pressure. The feed gas and the yield were analyzed by online gas chromatography (GC) equipped with a thermal conductivity detector (TCD). The conversion rate of NH_3_ (X_NH_3__) and the generation rate of H_2_ (r_H_2__) were calculated using the following formula:

$$X_{{NH}_{3}}(\%)=\frac{{{[V}_{N_{2}}]}_{out}/{{[V}_{{NH}_{3}}]}_{out}}{{{[V}_{N_{2}}]}_{out}/{{[V}_{{NH}_{3}}]}_{out}+0.5}\times 100\%$$

$$r_{H_{2}}(mmol/g_{cat}/min)=\frac{\frac{V_{{NH}_{3}}}{22.4}\times X_{{NH}_{3}}\times 1.5}{m_{cat}}$$

where [V_N2_]_out_, and [V_NH3_]_out_ are the volume percentage of N_2_ and NH_3_ in the effluent, respectively. V_NH3_ is the NH_3_ flow rate (mL/min), and m_cat_ is the mass of the catalyst (g).

## 4. Conclusions

In this study, Ce-MOF-derived CeO_2_ carriers were prepared by calcining Ce-BPDC, and then the obtained CeO_2_ was reduced in a H_2_/Ar atmosphere for different times (t = 0, 0.5, 1, 1.5, 2, 3, 4 h). Finally, a series of Ru-based catalysts were prepared by the impregnation method. The ammonia decomposition results showed that the performance of the Ru/CeO_2_-t was superior to that of the commercial CeO_2_-supported Ru catalyst. Under the condition of GHSV = 36,000 mL g_cat_^−1^ h^−1^, the ammonia conversion rate and hydrogen production rate of Ru/CeO_2_-4 h at 500 °C were 98.9% and 39.74 mmol g_cat_^−1^ min^−1^, respectively. Furthermore, the catalytic activity remained stable after continuous testing for 50 h. The XRD, SEM, and TEM results showed that Ru was well dispersed on the surface of CeO_2_-t. The Raman and XPS characterization results showed that, with the extension of the reduction time, the number of oxygen vacancies in these samples increased. This phenomenon can be attributed to the valence state transformation from Ce^4+^ to Ce^3+^ on the surface of CeO_2_ during the hydrogen reduction process, thereby inducing the generation of more O_V_. The results of the synchrotron radiation showed that an increase in oxygen vacancy concentration can enhance the interaction between the metal and the carrier. The DFT calculation determined that the rate-determining step of ammonia decomposition was the cleavage of the N-H bond, and the existence of oxygen vacancies can significantly reduce the reaction energy barrier of N-H cleavage, thereby improving the ammonia decomposition performance. These findings highlight a new idea for the design of ammonia decomposition catalysts and might open up new possibilities for the development of MOF-based catalysts in industrial application.

## Figures and Tables

**Table 1:** NH_3_ conversion of Ru/CeO_2_-t catalyst at GHSV = 12,000 mL g_cat_^−1^ h^−1^.

	T °C	375	400	425	450	475	500
NH_3_ Conv./% ^a^							
Ru/CeO_2_-C		7.87	16.86	33.47	52.66	73.75	85.16
Ru/CeO_2_-0 h		23.41	45.26	64.44	78.95	91.88	95.95
Ru/CeO_2_-0.5 h		30.24	55.79	73.53	86.24	92.95	96.99
Ru/CeO_2_-1 h		34.92	57.94	75.56	87.79	95.7	97.72
Ru/CeO_2_-1.5 h		38.85	61.37	78.57	89.77	97.11	99.01
Ru/CeO_2_-2 h		37.98	58.97	76	87.84	96.63	98.13
Ru/CeO_2_-3 h		42.86	64.91	81.04	92.19	98.22	99.90
Ru/CeO_2_-4 h		43.58	65.10	81.74	92.39	98.38	100.00

^a^ The calculation formula of NH_3_ conv./% is ${\;X}_{{NH}_{3}}(\%)=\frac{{\left[{NH}_{3}\right]}_{in}-{\left[{NH}_{3}\right]}_{out}}{{\left[{NH}_{3}\right]}_{out}}\times 100\%$, which is described in detail in Section 3.3.

## Data Availability

The data presented in this study are available upon request from the corresponding authors.

## Supplementary Materials

The following Supporting Information can be downloaded at https://www.mdpi.com/article/10.3390/molecules30112301/s1: Table S1: NH_3_ conversion of Ru/CeO_2_-C catalyst at GHSV = 12,000 mL g_cat_^−1^ h^−1^. Table S2: Comparison of the synthesized catalysts with Ru-based catalysts reported in the literature. Table S3: The Raman quantification results. Table S4: XPS quantitative results of CeO_2_-t and Ru/CeO_2_-t. Table S5: XPS quantitative results of CeO_2_-C and Ru/CeO_2_-C. Figure S1: The XRD profile of the Ce-BPDC (a) and the SEM images of the Ce-BPDC (b). Figure S2: N_2_ adsorption/desorption isotherms (a) and aperture distribution curve (b) of Ce-BPDC. Figure S3: The TGA curve of the Ce-BPDC. Figure S4: The XRD profile of the CeO_2_-t. Figure S5: The N_2_ adsorption isotherm of the derivative cerium oxide (a) and its corresponding catalyst (b). Figure S6: The SEM of the CeO_2-_0 h (a), CeO_2_-0.5 h (b), CeO_2_-1 h (c), CeO_2_-1.5 h (d), CeO_2_-2 h (e), CeO_2_-3 h (f). Figure S7: The TEM of the CeO_2_-t. Figure S8: The TEM and the corresponding EDS of Ru/CeO_2_-t. Figure S9: The AC-STEM of Ru/CeO_2_-0 h (a), Ru/CeO_2_-0.5 h (b), Ru/CeO_2_-1 h (c), Ru/CeO_2_-1.5 h (d), Ru/CeO_2_-2 h (e), Ru/CeO_2_-3 h(f), Ru/CeO_2_-4 h(g). Figure S10: Schematic diagram of a fixed-bed reactor. Figure S11: NH_3_ conversion diagram of Ru/CeO_2_-4 h catalyst, GHSV = 36,000 mL g_cat_^−1^ h^−1^. Figure S12. Stability test of Ru/CeO_2_-4 h catalyst, GHSV = 36,000 mL g_cat_^−1^ h^−1^. Figure S13: Raman spectra of CeO_2_-C (a) and Ru/CeO_2_-C (b). Figure S14: The Ru 3p_3/2_ XPS of Ru/CeO_2_-t. Figure S15: The (a) Ce 3d and (b) O 1s XPS of CeO_2_-t. Figure S16: The (a) Ce 3d and (b) O 1s XPS of CeO_2_-C, (c) Ce 3d and (d) O 1s XPS of Ru/CeO_2_-C. Figure S17: CO_2_-TPD curves for catalyst. Figure S18: XAS characterization results: (a) Ru K-edge XANES spectra of Ru/CeO_2_-t and (b) Fourier transform K3-weighted EXAFS spectra. Figure S19: Adsorption energy of NH_3_ on surfaces of CeO_2_, Ru/CeO_2_, and Ru/CeO_2_-Ov models. Figure S20: The reaction process of ammonia on Ru/CeO_2_ catalyst surface. Figure S21: The reaction process of ammonia on Ru/CeO_2_ catalyst surface. Figure S22: The reaction process of ammonia on Ru/CeO_2_-O_V_ catalyst surface. Figure S23: Ce 3d (a) and O 1s (b) spectra of catalyst Ru/CeO_2_-2 h before and after pretreatment. References [55,56,57,58,59,60,61,62,63,64,65,66,67,68,69,70,71,72,73,74,75,76] are cited in the supplementary materials.
