# Supplementary material for: Efficient Hydrogen Production from Ammonia Using Ru Nanoparticles on Ce-Based Metal–Organic Framework (MOF)-Derived CeO2 with Oxygen Vacancies
Source: Molecules. 2025 May 23;30(11):2301. doi: 10.3390/molecules30112301 (PMC12156145; doi:10.3390/molecules30112301)
Supplement: Supplementary file 1 [file molecules-30-02301-s001.zip › molecules-3567812-supplementary.pdf]

Supporting Information

Efficient hydrogen production from ammonia using Ru nanoparticles on Ce-MOF-derived CeO_2 with oxygen vacancies

Wenying Wu[1], Wenhao Yao[1], Yitong Liu[1], Senliang Xi[1,*] and Teng Zhang[1,*]

1 Key Laboratory of Cluster Science Ministry of Education, Beijing Key Laboratory of Photoelectronic/Electrophotonic Conversion Materials, Advanced Research Institute of Multidisciplinary Science, School of Chemistry and Chemical Engineering, Beijing Institute of Technology Beijing 100081, China; wwy15933833065@163.com (W.W.); ywh200012@163.com (W.Y.); m18735721812_1@163.com (Y.L.).

* Correspondence: xisenliang1996@163.com (S.X.), zhanghaiteng11@163.com (T.Z.)

Synthesis of Ce-MOFs and their derivative CeO_2

Ce-BPDC MOFs are synthesized by an improved hydrothermal method [55]. First, 0.548 g (1 mmol) ammonium cerium nitrate ($(NH_4)_2Ce(NO_3)_6$) was dissolved in 2 mL distilled water,denoted as solution A. 0.242 g 4-4' diphenyl dicarboxylic acid (H_2BPDC, 1 mmol) was dissolved in 6 mL N, N-dimethylformamide (DMF), denoted as solution B, slowly pour solution A into solution B, and then stirred at 100 °C for 15 min. After natural cooling, the yellow precipitate was obtained by centrifugation. Subsequently, the precipitate was washed 3 times with DMF, soaked overnight in acetone, and then dried at 80 °C for 2 h. Finally, the yellow Ce-BPDC product was obtained.

Ce-BPDC was calcined at 500 °C for 5 h to obtain CeO_2. Then the obtained CeO_2 was reduced at 500 °C in 5% H_2/A atmosphere for different times (t = 0, 0.5, 1, 1.5, 2, 3, 4 h), which was denoted as CeO_2-t.

Catalyst characterization

Powder X-ray diffractometer (PXRD), instrument model: Rigaku MiniFlex600. Instrument test conditions: pressure 40 kV, current 50 mA, X-ray radiation source Cu-Kα (λ = 0.154056 nm), 2θ = 10-90°, speed 10° min^{-1}.

Specific surface and aperture distribution analyzer, instrument model: Kubo X1000. Test condition: N_2, 77 K.

Field emission scanning electron microscope (SEM), instrument model: JEOL Model JSM-7500F. Instrument test conditions: pressure 40 kV.

Thermogravimetric (TG) analysis was performed on a METTLER Toledo thermogravimetric analyzer. Test conditions is heated from room temperature to 800 °C (ramping rate: 10 °C/min) in air (20 mL/min).

Transmission electron microscopy (TEM) images of the sample were performed on a JEOL JEM-F200 electron microscope with an accelerated voltage of 200 kV.

Spherical Aberration Transmission of Condenser Lens (AC-STEM), instrument model: Thermo Scientific spectra 300.

The energy-dispersive X-ray (EDX) maps were obtained with a Super-X G2 detector.

Inductively coupled plasma optical emission spectroscopy (ICP-OES) analysis of the Ru content was performed on a Horiba Jobin Yvon Activa instrument.

Raman spectroscopy measurements were performed using the Horiba Scientific LabRAM HR Evolution Raman spectrometer, with a 405 nm laser as the excitation source for Raman spectroscopy analysis.

X-ray photoelectron spectroscopy (XPS) measurements were made using an American ThermoFischer, ESCALAB 250Xi spectrometer equipped with an Al Kα (hv=1486.6 eV) X-ray source.

Carbon Dioxide Temperature Programmed Desorption (CO_2-TPD) experiments were carried out on the analytical decar automatic Chemisorption analyzer (ChemBET Pulsar TPR/TPD). The 100 mg sample was weighed and placed in the reaction tube. The sample was heated at 10 °C/min from room temperature to 500 °C for drying pre-treatment. The sample was purged with helium (30-50 mL/min) for 1 h, cooled to 50 °C, and then injected with carbon dioxide and helium 10% CO_2/He mixture (30-50 mL/min) for 1 h until saturated. Switching air flow (30-50 mL/min) to purge for 1 h to remove the weakly physically adsorbed CO_2 on the surface. Finally, the desorption rate was increased to 800 °C at 10 °C/min in He atmosphere,

and the exhaled gas was detected by Thermal Conductivity Detector (TCD).

Synchrotron radiation X-ray absorption fine structure (XAFS), instrument model: Spring8 BL14B2. Prior to the test, the samples were reduced in situ at 200 °C for 2 h under 2.0×10^4 Pa H_2 pressure in a pretreatment chamber.

Density Functional Theory (DFT)

All DFT calculations were performed using the Vienna Ab initio Simulation Package (VASP) [56]. The projector augmented wave (PAW) [57] pseudopotential with the PBE [58] generalized gradient approximation (GGA) exchange correlation function was utilized in the computations. The cutoff energy of the plane waves basis set was 520 eV and a MonkhorstPack mesh of 2 × 2 × 1 was used in K-sampling. All structures were spin polarized and all atoms were fully relaxed with the energy convergence tolerance of 10^{-5} eV per atom, and the final force on each atom was < 0.05 eV $Å^{-1}$.

The equilibrium lattice constants of CeO_2 unit cell were optimized. We then use it to construct a CeO_2 (111) surface model. During structural optimizations, a 3 × 3 × 1 k-point grid in the Brillouin zone was used for k-point sampling, and the bottom two atomic layers were fixed while the others were allowed to relax. The Ru/CeO_2 (111) structures had been established using the CeO_2 (111) surface and Ru nano-cluster with the lattice parameters (a = 11.5983 Å, b = 11.5983 Å, c = 27.8917 Å). The Ru-CeO_2 (111) structures with 3-layer of Ce-O-Ce-O layers and 13 Ru atoms include the 93 atoms. The Ru nano-cluster is composed of 13 Ru atoms, and it had been optimized using DFT methods. Ru-CeO_2 (111)-O_V structure is established, and an O atom is randomly removed around the Ru cluster on the Ru-CeO_2 (111) structure to form an O vacancy.

Finally, the adsorption energies(Eads) were calculated as Eads = Ead/sub - Ead - Esub, where Ead/sub, Ead, and Esub are the total energies of the optimized adsorbate/substrate system, the adsorbate in the structure, and the clean substrate, respectively. The free energy was calculated using the equation:

$$G = Eads + ZPE - TS$$

where G, Eads, ZPE and TS are the free energy, total energy from DFT calculations, zero point energy and entropic contributions, respectively.

The characterization information of Ce-MOF

Ce-BPDC was selected as the precursor of catalyst synthesis and its crystal structure was analyzed by powder X-ray diffraction (PXRD) technique (Figure S1a). The results showed that the obtained Ce-BPDC exhibited the same crystal structure as in the literature. The BET specific surface area and pore size calculated according to N_2 adsorption/desorption isotherm (Figure S2) were 1502 m^2 g^{-1} and 1.0 nm, respectively. Scanning electron microscopy (SEM) (Figure. S1b) was used to observe the surface morphology of Ce-BPDC as cubic particles.

The thermal stability of Ce-BPDC in an air atmosphere was investigated by thermogravimetry, and the results were shown in Figure S3. Ce-BPDC rapidly losed weight at 350 °C, and the weight did not change anymore after 400 °C, indicating that the organic skeleton had been sufficiently removed and Ce-BPDC was completely converted to CeO_2.

Table of Contents

Table S1. NH_3 conversion of Ru/CeO_2-C catalyst at GHSV = 12000 mL g_{cat}^{-1} h^{-1}. 5

Table S2. Comparison of the synthesized catalysts with Ru based catalysts reported in the literature. 5

Table S3. The Raman quantification results 6

Table S4. XPS quantitative results of CeO_2-t and Ru/CeO_2-t. 6

Table S5. XPS quantitative results of CeO_2-C and Ru/CeO_2-C. 6

Figure S1. The XRD profile of the Ce-BPDC (a) and the SEM images of the Ce-BPDC (b). 7

Figure S2. N_2 adsorption/desorption isotherms (a) and aperture distribution curve (b) of Ce-BPDC. 7

Figure S3. The TGA curve of the Ce-BPDC. 7

Figure S4. The XRD profile of the CeO_2-t. 8

Figure S5. The N_2 adsorption isotherm of the derivative cerium oxide (a) and its corresponding catalyst (b). 8

Figure S6. The SEM of the CeO_2-0 h (a)、CeO_2-0.5 h (b)、CeO_2-1 h (c)、CeO_2-1.5 h (d)、CeO_2-2 h (e)、CeO_2-3 h(f)、CeO_2-4 h(g). 9

Figure S7. The TEM of the CeO_2-0 h (a)、CeO_2-0.5 h (b)、CeO_2-1 h (c)、CeO_2-1.5 h (d)、CeO_2-2 h (e)、CeO_2-3 h(f)、CeO_2-4 h(g). 10

Figure S8. The TEM and the corresponding EDS of Ru/CeO_2-0 h (a)、Ru/CeO_2-0.5 h (b)、Ru/CeO_2-1 h (c)、Ru/CeO_2-1.5 h (d)、Ru/CeO_2-2 h (e)、Ru/CeO_2-3 h(f)、Ru/CeO_2-4 h(g). 12

Figure S9. The AC-STEM of Ru/CeO_2-0 h (a)、Ru/CeO_2-0.5 h (b)、Ru/CeO_2-1 h (c)、Ru/CeO_2-1.5 h (d)、Ru/CeO_2-2 h (e)、Ru/CeO_2-3 h(f)、Ru/CeO_2-4 h(g). 12

Figure S10. Schematic diagram of a fixed bed reactor. 13

Figure S11. NH_3 conversion diagram of Ru/CeO_2-4 h catalyst, GHSV = 36000 mL g_{cat}^{-1} h^{-1}. 14

Figure S12. Stability test of Ru/CeO_2-4 h catalyst, GHSV = 36000 mL g_{cat}^{-1} h^{-1}. 14

Figure S13. Raman spectra of CeO_2-C (a) and Ru/CeO_2-C (b). 15

Figure S14. The Ru $3p_{3/2}$ XPS of Ru/CeO_2-t. 16

Figure S15. The (a) Ce 3d and (b) O 1s XPS of CeO_2-t. 17

Figure S16. The (a) Ce 3d and (b) O 1s XPS of CeO_2-C, (c) Ce 3d and (d) O 1s XPS of Ru/CeO_2-

C. 18

Figure S17. CO_2-TPD curves for catalyst. 17

Figure S18. XAS characterization results: (a) Ru K-edge XANES spectra of Ru/CeO_2-t and (b) Fourier transform K3-weighted EXAFS spectra. 18

Figure S19. CeO_2, Ru/CeO_2 and Ru/CeO_2-O_V models. 18

Figure S20. Adsorption energy of NH_3 on surfaces of CeO_2, Ru/CeO_2 and Ru/CeO_2-Ov models. 18

Figure S21. The reaction process of ammonia on Ru/CeO_2 catalyst surface. 19

Figure S22. The reaction process of ammonia on Ru/CeO_2-O_V catalyst surface. 20

Figure S23. Ce 3d (a) and O 1s (b) spectra of catalyst Ru/CeO_2-2 h before and after pretreatment. 21

Table S1. NH_3 conversion of Ru/CeO_2-C catalyst at GHSV = 12000 mL g_{cat}^{-1} h^{-1}.

T °C / NH_3 conv./%[a]	375	400	425	450	475	500
Ru/CeO_2-C	7.87	16.86	33.47	52.66	73.75	85.16
Ru/CeO_2-C-2 h	9.96	20.28	35.98	53.21	77.41	87.95
Ru/CeO_2-C-4 h	12.23	29.89	46.32	67.66	80.95	90.11

Results in Table S1 indicate that loading Ru on reduced commercial CeO_2 fails to substantially enhance the catalytic performance.

Table S2. Comparison of the synthesized catalysts with Ru based catalysts reported in the literature.

catalyst	Ru/wt %	T/°C	GHSV/mL g_{cat}^{-1} h^{-1}	NH_3 conv./%	H_2 formation rate/mmol g_{cat}^{-1} min^{-1}	Ref.
Ru/CeO_2-4 h	4.19	475	36000	97.04	38.99	This work
		500	36000	98.91	39.74	
Ru/$La_{0.8}Sr_{0.2}AlO_3$	3	500	30000	71.6	24.0	[12]
K-Ru/MCM-41	5	500	15000	87.8	29.4	[60]
Ru/Ba-ZrO_2	3	500	30000	53.0	17.8	[61]
Ru/Y_2O_3	5	500	30000	99.9	30	[62]
Ru/BCY-10	2	500	6000	61	20.4	[63]
Ru/La-ZrO_2	3	500	4000	75.0	25.1	[33]
Ru/MgO	5	500	36000	91.0	30.1	[26]
Ru/Pr_2O_3	5	475	30000	67.5	22.6	[64]
Ru/Al_2O_3	2.1	500	30000	21.0	7.0	[65]
1Ru/Al_2O_3	1	450	30000	10	4.5	[66]
Ru/Cr_2O_3	5	500	30000	25	8.4	[67]
Ru/CeO_2	2	500	3607	99.9	6.7	[68]
10Ni_2Ru/CeO_2	2	500	3660	99.2	16.6	[69]
Ru/La_2O_3-Al_2O_3	2	500	10000	72.1	8.05	[70]
Ru/La_2O_3-700-i	4.8	450	18000	58.2	11.7	[71]
Ru/γ-Al_2O_3	5	550	30000	73.7	23.5	[72]
Ru/La(10)-Al_2O_3	2	550	10000	81	8.3	[73]
Ru/ZrO_2	5	550	15000	77	25.8	[72]
Ru/CeO_2	7	400	6000	73	4.89	[74]
Pd/CNTs	5.1	500	30000	5	1.43	[13]
Ru/AC	5	550	30000	84.7	26	[75]
Ru/GA	13.6	450	30000	71.5	21.9	[76]

Table S3. The Raman quantification results.

Samples	I_D/I_{F2g}	Samples	I_D/I_{F2g}
CeO_2-0 h	0.023	Ru/CeO_2	0.091
CeO_2-0.5 h	0.062	Ru/CeO_2-0.5 h	0.109
CeO_2-1 h	0.066	Ru/CeO_2-1 h	0.156
CeO_2-1.5 h	0.072	Ru/CeO_2-1.5 h	0.179
CeO_2-2 h	0.068	Ru/CeO_2-2 h	0.171
CeO_2-3 h	0.091	Ru/CeO_2-3 h	0.185
CeO_2-4 h	0.093	Ru/CeO_2-4 h	0.188

Table S4. XPS quantitative results of CeO_2-t and Ru/CeO_2-t.

Samples	$Ce^{3+}/(Ce^{3+} + Ce^{4+})$ (%)	$O_V/(O_V + O_L + O_C)$ (%)
CeO_2-0 h	16.4	29.17
CeO_2-0.5 h	17.12	29.5
CeO_2-1 h	17.2	29.69
CeO_2-1.5 h	17.26	31.55
CeO_2-2 h	19.98	32.46
CeO_2-3 h	23.23	33.6
CeO_2-4 h	25.53	35.47
Ru/CeO_2-0 h	22.02	38.68
Ru/CeO_2-0.5 h	22.57	40.54
Ru/CeO_2-1 h	23.69	41.39
Ru/CeO_2-1.5 h	27.63	41.9
Ru/CeO_2-2 h	28	42
Ru/CeO_2-3 h	31.82	43.19
Ru/CeO_2-4 h	31.93	44.64

Table S5. XPS quantitative results of CeO_2-C and Ru/CeO_2-C.

Samples	$Ce^{3+}/(Ce^{3+} + Ce^{4+})$ (%)	$O_V/(O_V + O_L + O_C)$ (%)
CeO_2-C	11.3	16.7
CeO_2-C-2 h	12.4	17.8
CeO_2-C-4 h	13.5	18.3
Ru/CeO_2-C	18.1	22.4
Ru/CeO_2-C-2 h	19.3	23.4
Ru/CeO_2-C-4 h	22.6	26.8

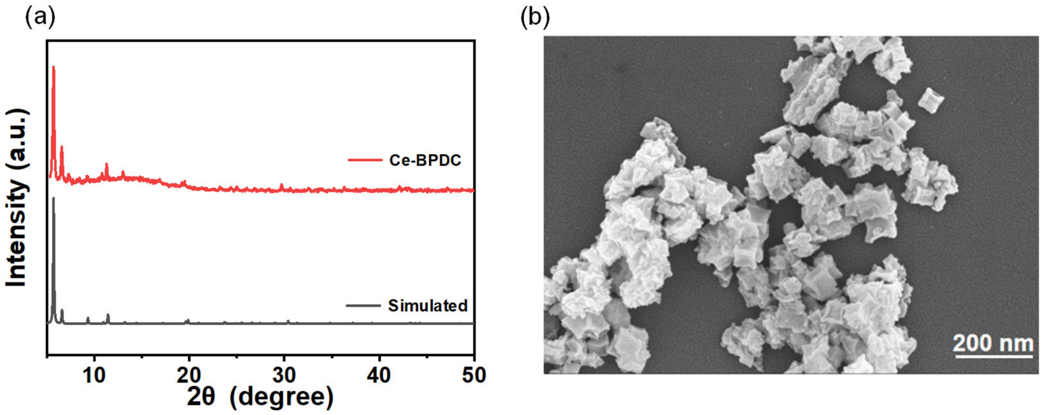

Figure S1. The XRD profile of the Ce-BPDC (a) and the SEM images of the Ce-BPDC (b).

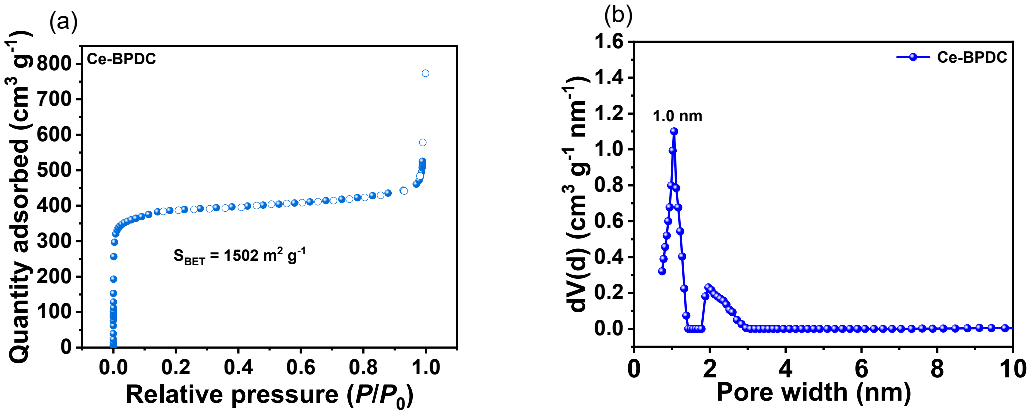

Figure S2. N_2 adsorption/desorption isotherms (a) and aperture distribution curve (b) of Ce-BPDC.

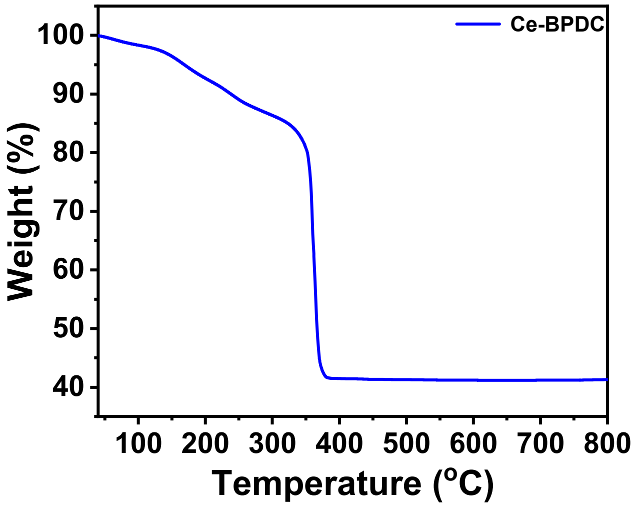

Figure S3. The TGA curve of the Ce-BPDC.

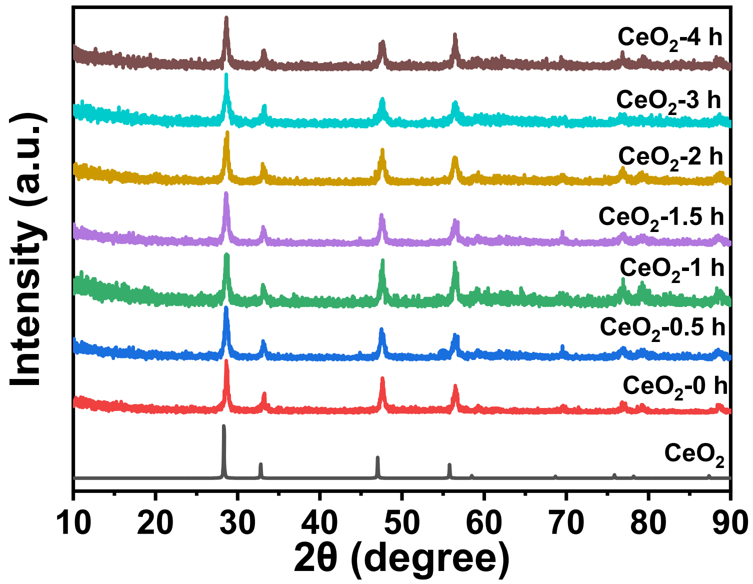

Figure S4. The XRD profile of the CeO_2-t.

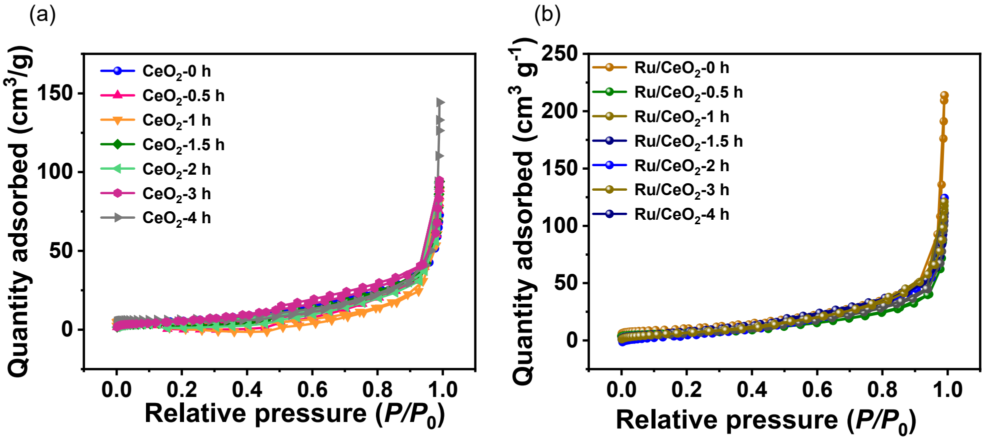

Figure S5. The N_2 adsorption isotherm of the derivative cerium oxide (a) and its corresponding catalyst (b).

The specific surface area is not significantly different from that of regular CeO_2 [59], as a calcination temperature of 500 °C was chosen in this study. When the calcination temperature exceeds the thermal stability threshold of MOF, the local high temperature generated by the decomposition of organic ligands can lead to the collapse of the porous structure. The rapid migration of oxygen atoms during calcination also accelerates surface reconstruction, eliminating the porous structure. Therefore, the structural guidance advantage of MOF under high-temperature calcination is offset by the intrinsic surface reconstruction behavior of CeO_2. Thus, the difference in specific surface area is not significant.

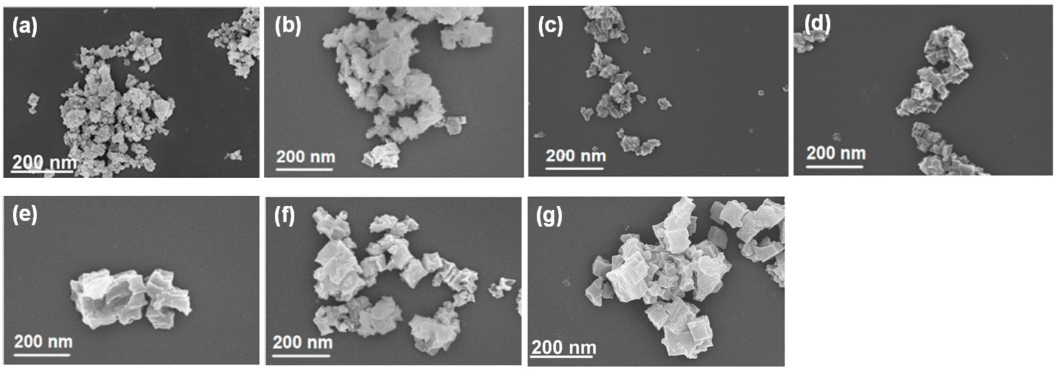

Figure S6. The SEM of the CeO_2 (a)、CeO_2-0.5 h (b)、CeO_2-1 h (c)、CeO_2-1.5 h (d)、CeO_2-2 h (e)、CeO_2-3 h(f)、CeO_2-4 h(g).

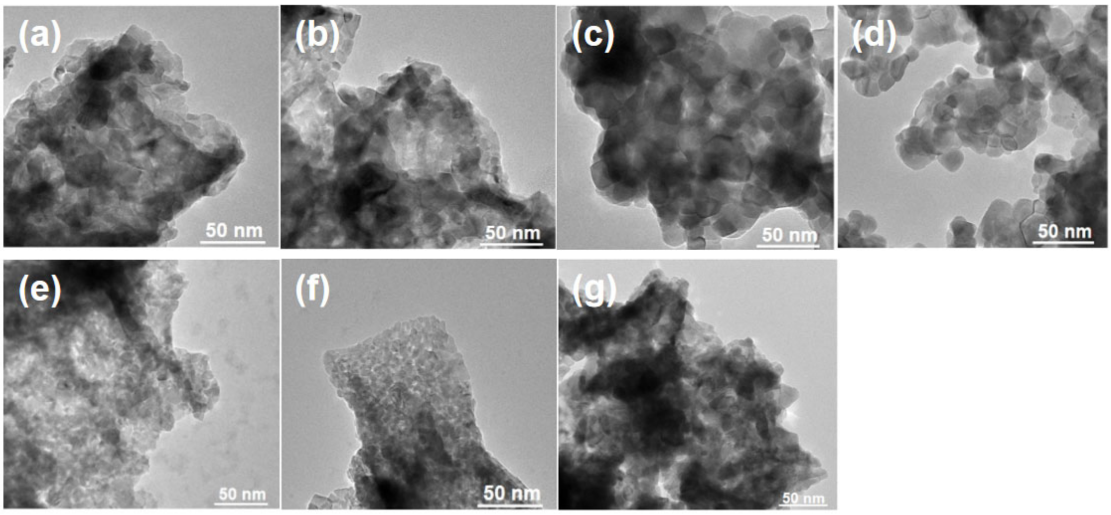

Figure S7. The TEM of the CeO_2-0 h(a)、CeO_2-0.5 h (b)、CeO_2-1 h (c)、CeO_2-1.5 h (d)、CeO_2-2 h (e)、CeO_2-3 h(f)、CeO_2-4 h(g).

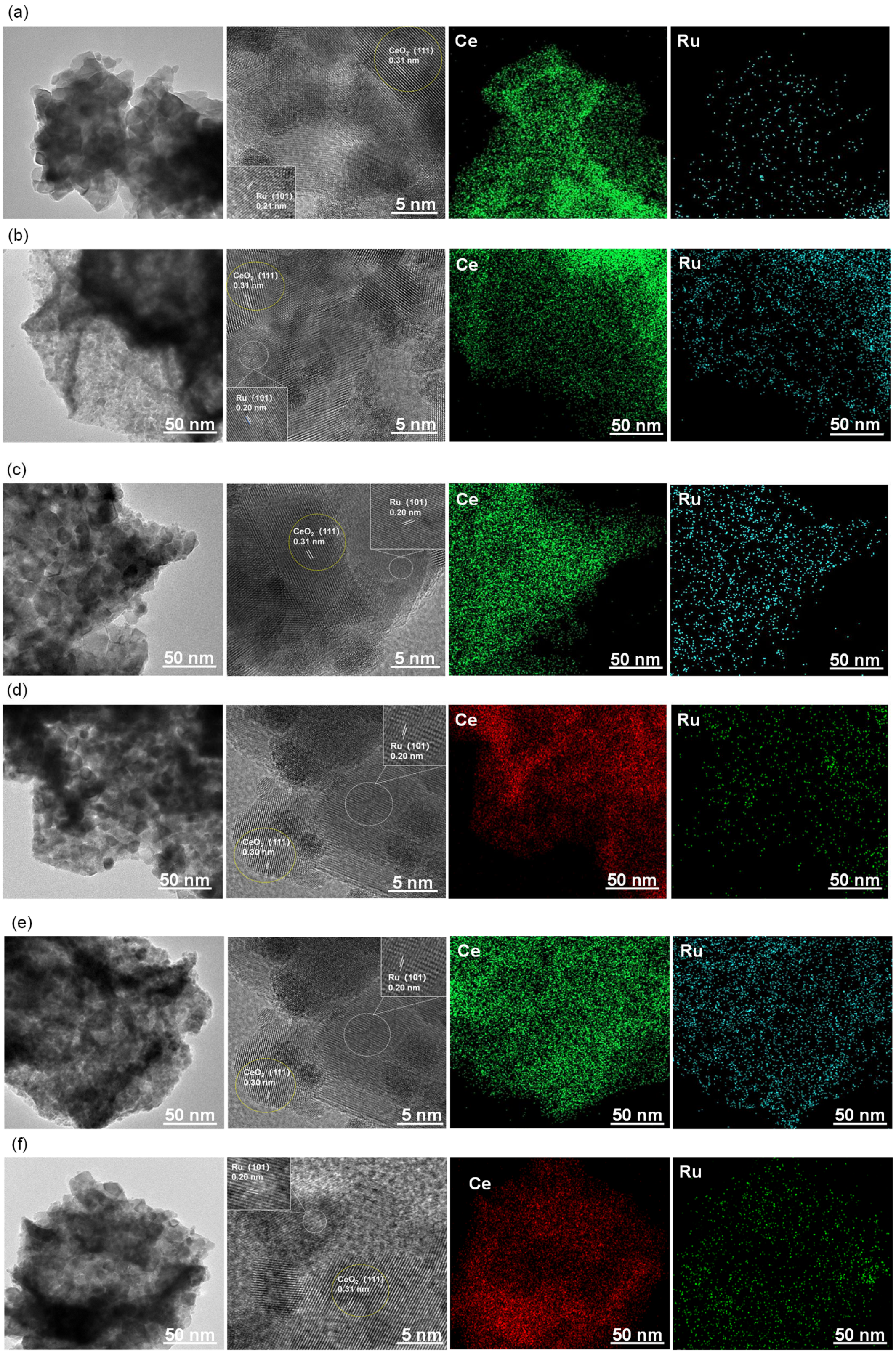

(a)
CeO2 (111)
0.31 nm
Ru (101)
0.21 nm
5 nm
Ce
Ru
(b)
50 nm
CeO2 (111)
0.31 nm
Ru (101)
0.20 nm
5 nm
Ce
50 nm
Ru
50 nm
(c)
50 nm
Ru (101)
0.20 nm
CeO2 (111)
0.31 nm
5 nm
Ce
50 nm
Ru
50 nm
(d)
50 nm
Ru (101)
0.20 nm
CeO2 (111)
0.30 nm
5 nm
Ce
50 nm
Ru
50 nm
(e)
50 nm
Ru (101)
0.20 nm
CeO2 (111)
0.30 nm
5 nm
Ce
50 nm
Ru
50 nm
(f)
50 nm
Ru (101)
0.20 nm
CeO2 (111)
0.31 nm
5 nm
Ce
50 nm
Ru
50 nm

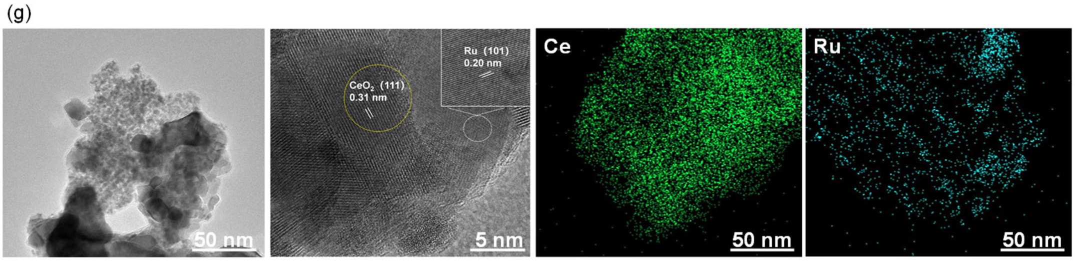

Figure S8. The TEM and the corresponding EDS of Ru/CeO_2-0 h (a)、Ru/CeO_2-0.5 h (b)、Ru/CeO_2-1 h (c)、Ru/CeO_2-1.5 h (d)、Ru/CeO_2-2 h (e)、Ru/CeO_2-3 h(f)、Ru/CeO_2-4 h(g).

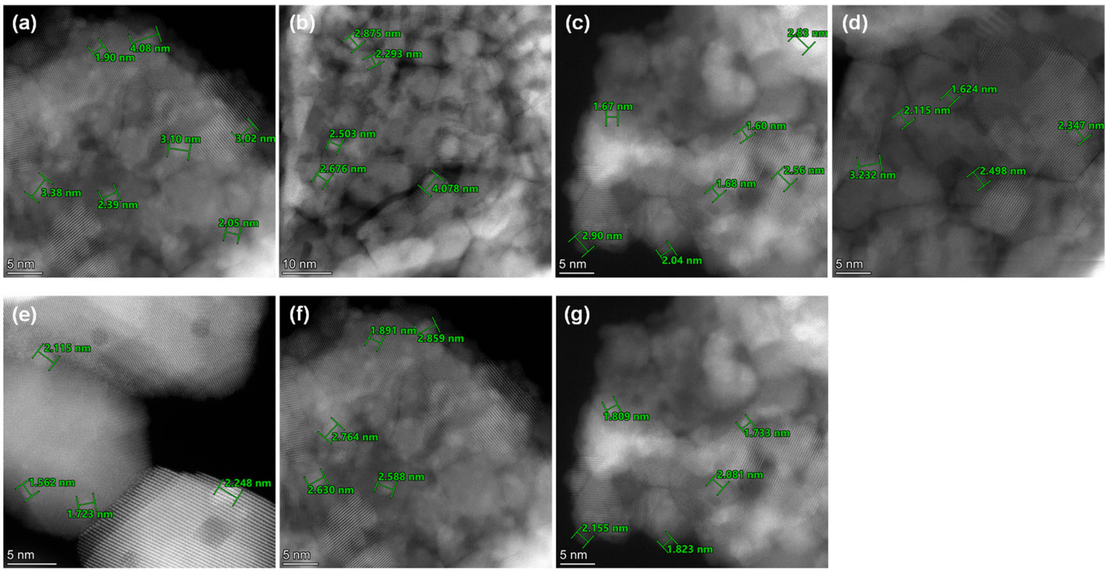

Figure S9. The AC-STEM of Ru/CeO_2-0 h (a)、Ru/CeO_2-0.5 h (b)、Ru/CeO_2-1 h (c)、Ru/CeO_2-1.5 h (d)、Ru/CO_2-2 h (e)、Ru/CeO_2-3 h(f)、Ru/CeO_2-4 h(g).

Due to the small load of Ru and the limited number of particles measured, there may be some uncertainty in the statistical results of the particle size distribution. Nevertheless, according to the existing data, the particle size of Ru is mainly concentrated in the range of 1.5-4 nm.

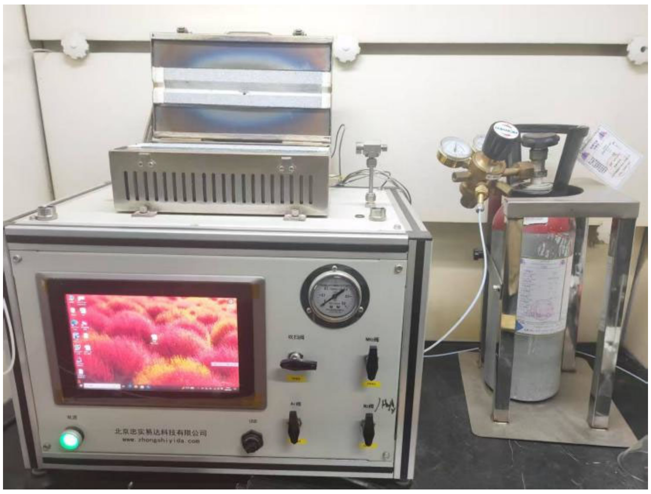

Figure S10. Schematic diagram of a fixed bed reactor.

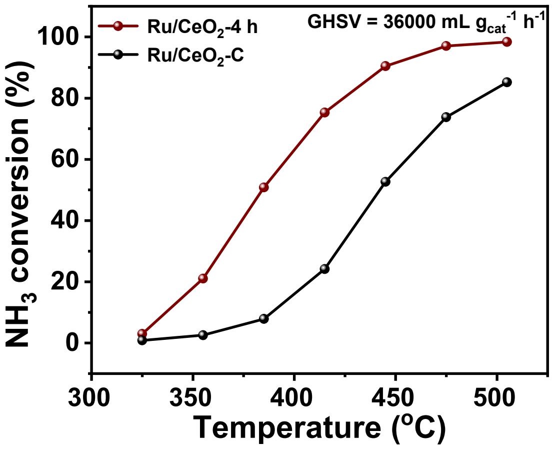

Figure S11. NH_3 conversion diagram of Ru/CeO_2-4 h catalyst, GHSV = 36000 mL g_{cat}^{-1} h^{-1}.

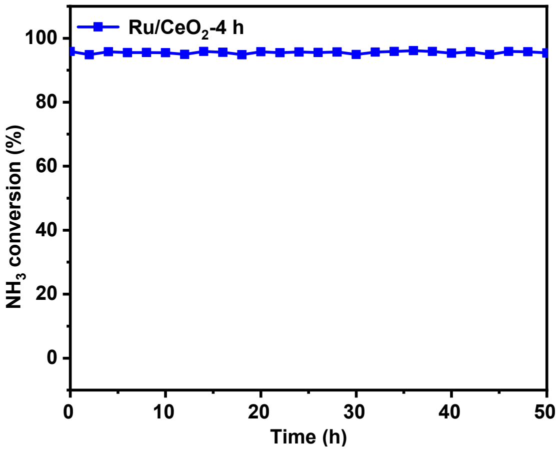

Figure S12. Stability test of Ru/CeO_2-4 h catalyst, GHSV = 36000 mL g_{cat}^{-1} h^{-1}.

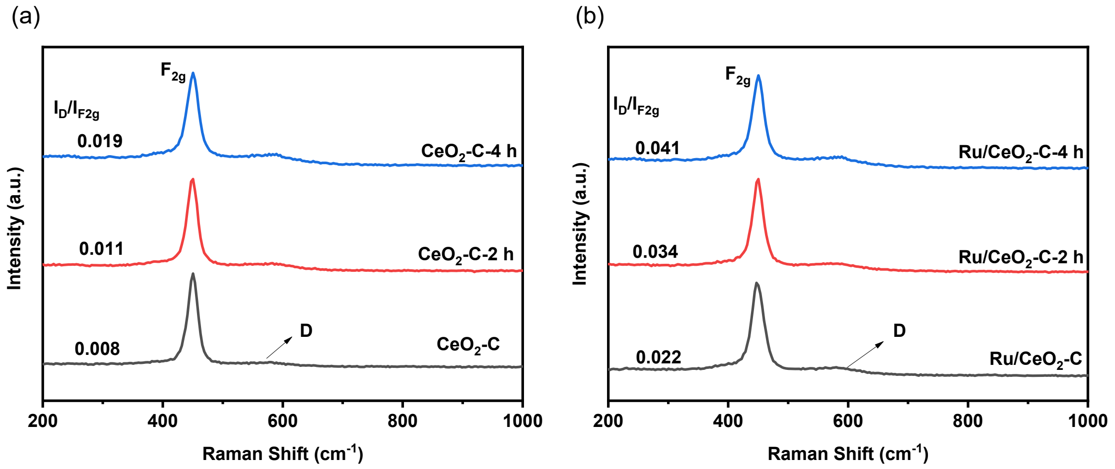

Figure S13. Raman spectra of CeO_2-C (a) and Ru/CeO_2-C (b).

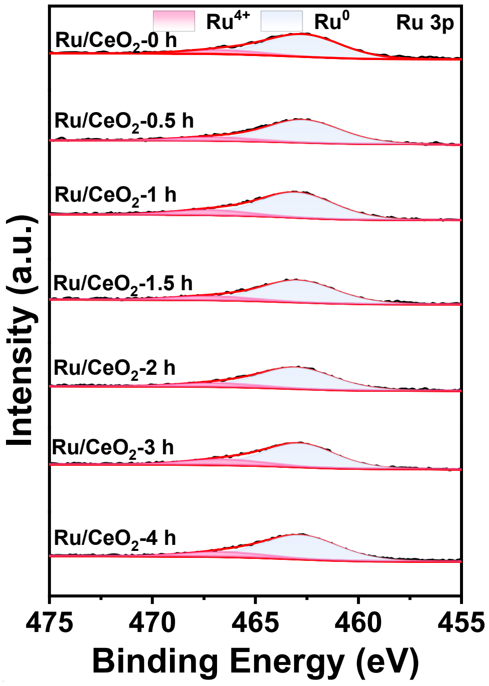

Figure S14. The Ru $3p_{3/2}$ XPS of Ru/CeO_2-t.

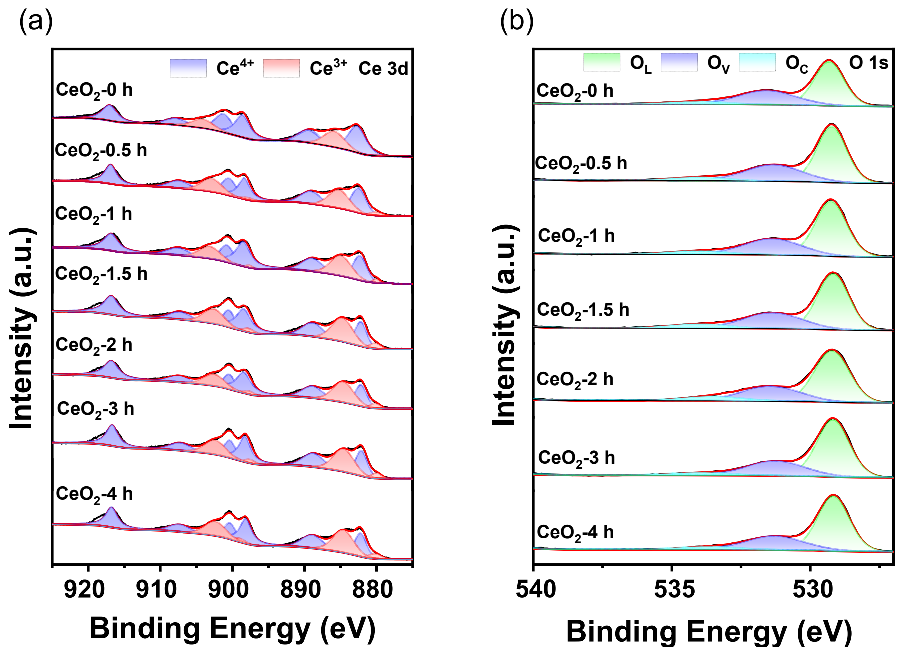

Figure S15. The (a) Ce 3d and (b) O 1s XPS of CeO_2-t.

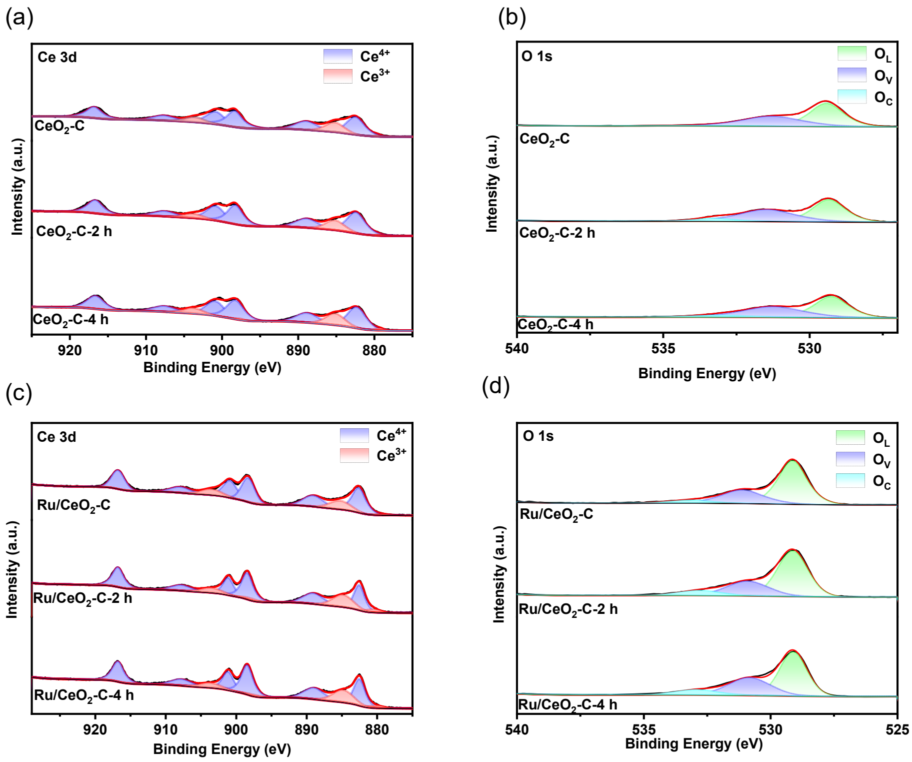

Figure S16. The (a) Ce 3d and (b) O 1s XPS of CeO_2-C, (c) Ce 3d and (d) O 1s XPS of Ru/CeO_2-C

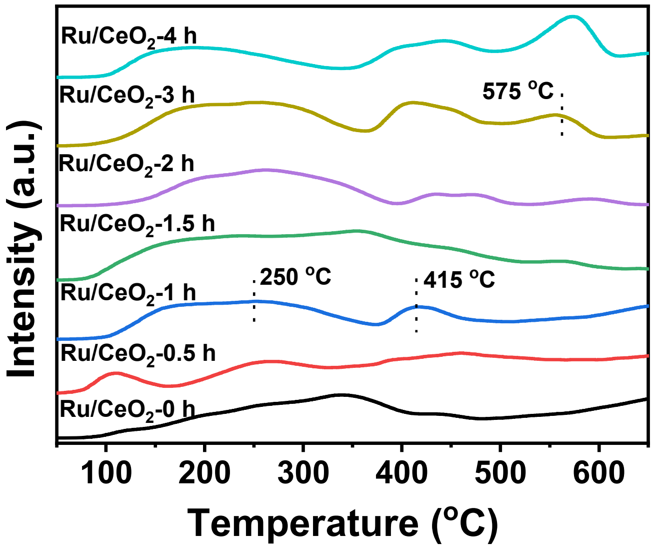

Figure S17. CO_2-TPD curves for catalyst.

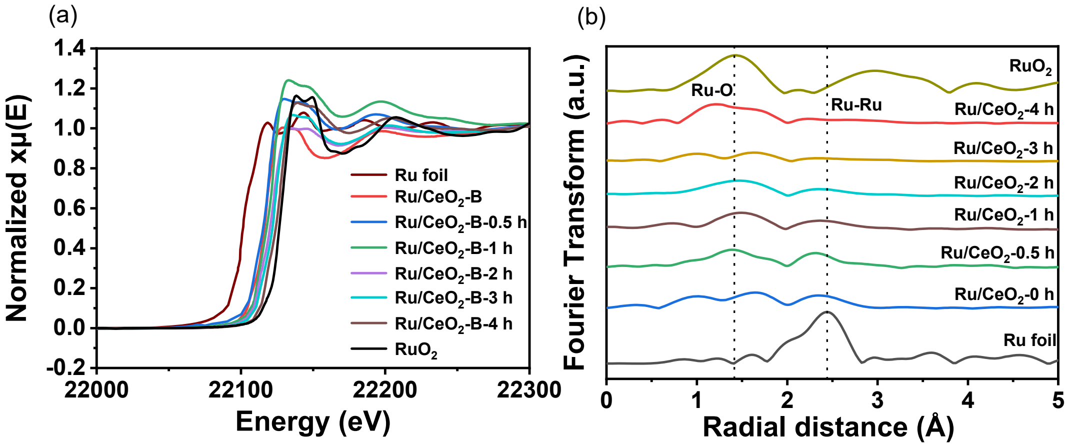

Figure S18. XAS characterization results: (a) Ru K-edge XANES spectra of Ru/CeO_2-t and (b) Fourier transform K3-weighted EXAFS spectra.

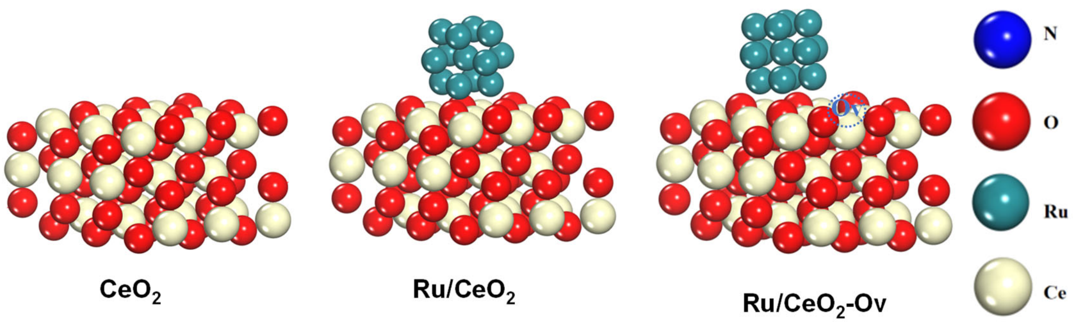

Figure S19. CeO_2, Ru/CeO_2 and Ru/CeO_2-Ov models.

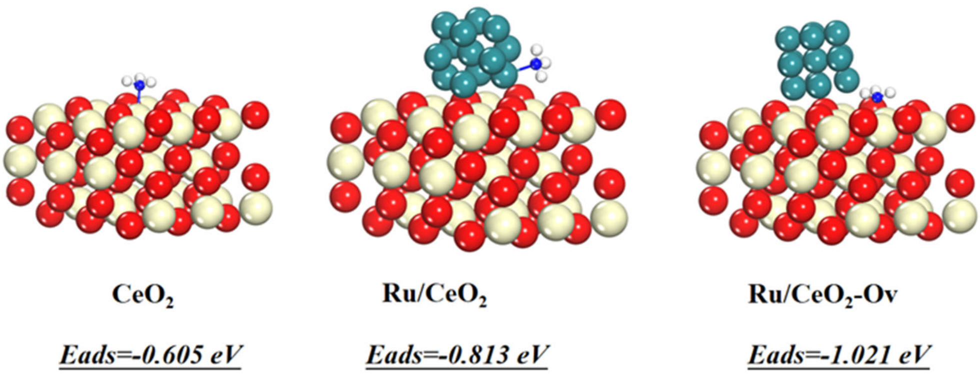

Figure S20. Adsorption energy of NH_3 on surfaces of CeO_2, Ru/CeO_2 and Ru/CeO_2-Ov models.

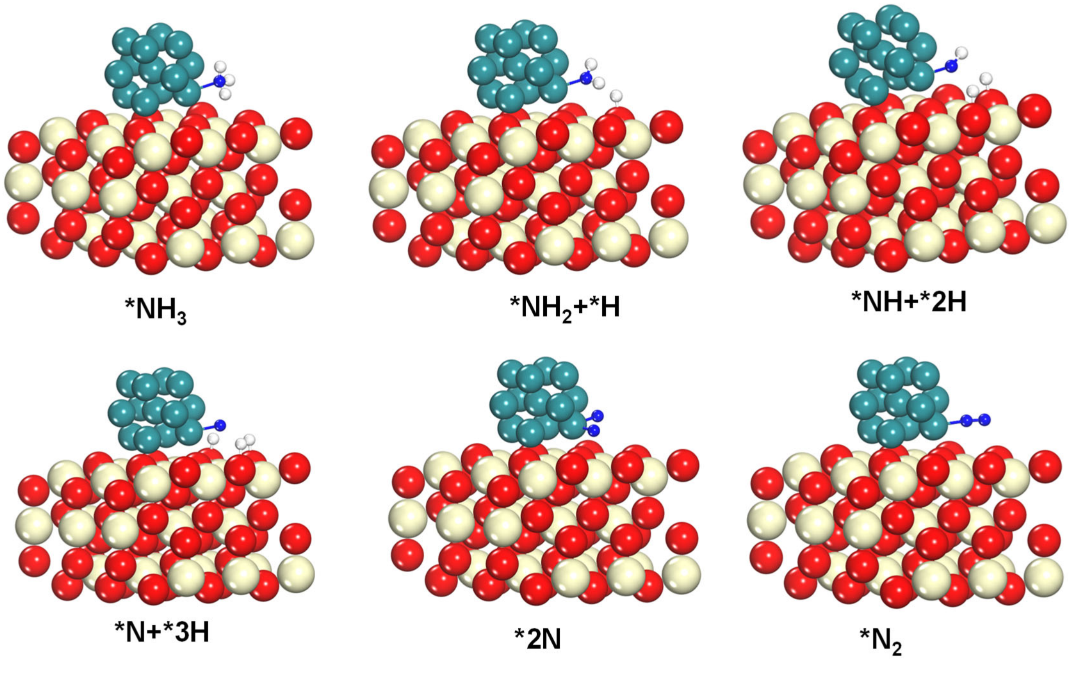

Figure S21. The reaction process of ammonia on Ru/CeO_2 catalyst surface.

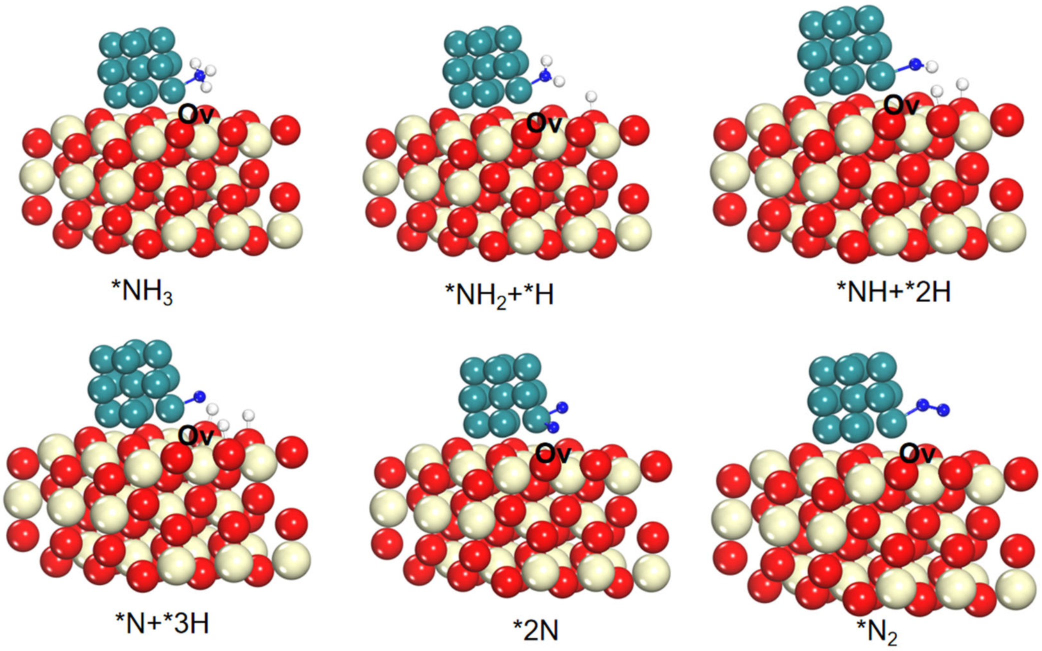

Figure S22. The reaction process of ammonia on Ru/CeO_2-Ov catalyst surface.

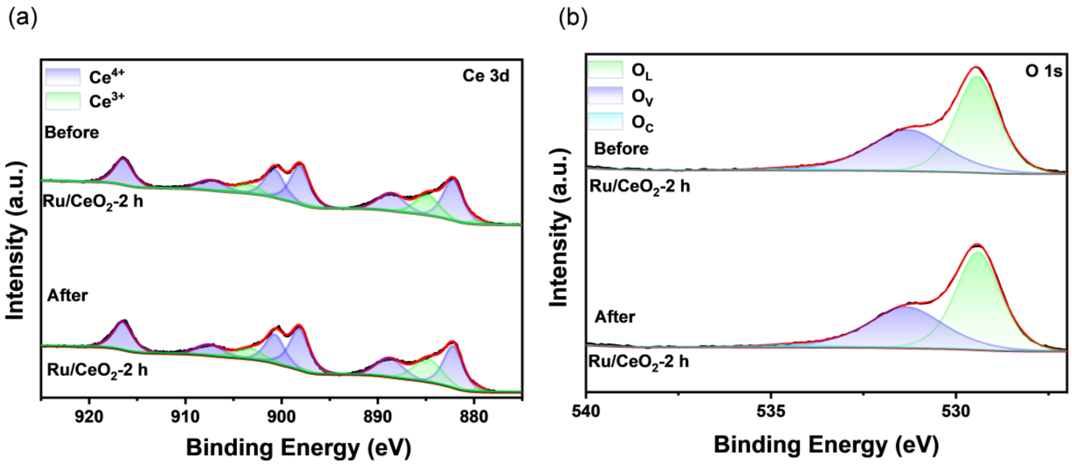

Figure S23. Ce 3d (a) and O 1s (b) spectra of catalyst Ru/CeO_2-2 h before and after pretreatment.

As shown in the figure, there was no significant change in the relative concentration of Ce^{3+} and O_V before and after catalyst reduction, indicating that the oxygen vacancy on the surface of the catalyst was not affected by the pre-reduction treatment at 200 °C.
